# Oxygen-independent disulfide bond formation in VEGF-A and CA9

**DOI:** 10.1016/j.jbc.2021.100505

**Published:** 2021-03-03

**Authors:** Fiana Levitin, Sandy Che-Eun S. Lee, Stephanie Hulme, Ryan A. Rumantir, Amy S. Wong, Marmendia R. Meester, Marianne Koritzinsky

**Affiliations:** 1 Princess Margaret Cancer Center, University Health Network, Toronto, Canada; 2 Institute of Medical Science, University of Toronto, Toronto, Canada; 3 Department of Radiation Oncology, University of Toronto, Toronto, Canada; 4 Department of Medical Biophysics, University of Toronto, Toronto, Canada

**Keywords:** carbonic anhydrase 9 (CA9), vascular endothelial growth factor (VEGF), low-density lipoprotein receptor (LDLR), protein folding, disulfide, glycosylation, endoplasmic reticulum (ER), hypoxia, tumor microenvironment, CA9, carbonic anhydrase 9, DTT, dithiothreitol, ER, endoplasmic reticulum, Flu-HA, influenza hemagglutinin, LDLR, low-density lipoprotein receptor, NEM, N-ethylmaleimide, UPR, unfolded protein response, VEGF, vascular endothelial growth factor

## Abstract

Low levels of oxygen (hypoxia) occurs in many (patho)physiological situations. Adaptation to hypoxia is in part mediated by proteins expressed in the extracellular space that mature in the endoplasmic reticulum (ER) prior to traversing the secretory pathway. The majority of such ER cargo proteins require disulfide bonds for structural stability. Disulfide bonds are formed co- and posttranslationally in a redox relay that requires a terminal electron acceptor such as oxygen. We have previously demonstrated that some ER cargo proteins such as low-density lipoprotein receptor (LDLR) and influenza hemagglutinin (Flu-HA) are unable to complete disulfide bond formation in the absence of oxygen, limiting their ability to pass ER quality control and their ultimate expression. Here, using radioactive pulse-chase immunoprecipitation analysis, we demonstrate that hypoxia-induced ER cargo proteins such as carbonic anhydrase 9 (CA9) and vascular endothelial growth factor A (VEGF-A) complete disulfide bond formation and mature with similar kinetics under hypoxia and normoxia. A global *in silico* analysis of ER cargo revealed that hypoxia-induced proteins on average contain fewer free cysteines and shorter-range disulfide bonds in comparison to other ER cargo proteins. These data demonstrate the existence of alternative electron acceptors to oxygen for disulfide bond formation *in cellulo*. However, the ability of different proteins to utilize an oxygen-independent pathway for disulfide bond formation varies widely, contributing to differential gene expression in hypoxia. The superior ability of hypoxia-induced proteins such as VEGF-A and CA9 to mature in hypoxia may be conferred by a simpler disulfide architecture.

Low levels of oxygen (hypoxia) occur during embryonic development and in pathologies such as wounds, ischemia and in solid tumors (1). The presence of tumor hypoxia confers resistance to conventional chemo- and radiotherapy (2). Cells under hypoxic stress alter their behavior through the upregulation of several proteins. These proteins are regulated at an epigenetic (3, 4), transcriptional (5) and translational (6) level. Most notably, the HIF family of hypoxia-inducible transcription factors are stabilized and activated to induce genes that contribute to metabolic adaptation. Our group and others have identified that hypoxia also readily activates the unfolded protein response (UPR) in mammalian cells (7, 8, 9). Three endoplasmic reticulum (ER) stress sensors are activated by the accumulation of unfolded or misfolded proteins in the ER, leading to translational repression and transcriptional upregulation of proteins involved in protein maturation, autophagy, and redox homeostasis (2, 10, 11, 12). Both HIF and UPR contribute to hypoxia tolerance and malignant cancer phenotypes (2).

These aggressive phenotypes are in part mediated by induction of secreted proteins such as vascular endothelial growth factor A (VEGF-A) that stimulate angiogenesis (13), and membrane-bound proteins such as carbonic anhydrase 9 (CA9) that contribute to cellular pH homeostasis (5, 14, 15). VEGF-A and CA9 are induced by HIF and UPR during hypoxia through both transcriptional and translational mechanisms, but little is known about the oxygen dependencies of protein maturation, which ultimately affects expression in the extracellular space.

Proteins that are destined for the cell membrane or secretion enter the ER cotranslationally before proceeding through the secretory pathway (16). The ER serves as a folding and maturation compartment for these “ER cargo” proteins. ER-localized co- and posttranslational modifications include N-linked glycosylation and glycan modifications, disulfide bond formation, and isomerization (16). Activation of the UPR by hypoxia suggests that one or more cellular processes in the ER are oxygen-dependent. While glycan processing has been identified as an oxygen-independent process (8), disulfide bond formation represents a net oxidation that requires a terminal oxidant (16, 17). Disulfide bonds are introduced between cysteine residues of proteins by protein disulfide isomerase (PDI) family members through the donation of its disulfide bond in the CXXC catalytic motif (16, 17). PDIs can also rearrange (isomerize) disulfide bonds in their clients. To support multiple cycles of disulfide bond formation, PDIs must be reoxidized, typically by the ER oxidoreductase flavoproteins ERO1-Lα or ERO1-Lß (18, 19, 20) that are in turn reoxidized by another protein or oxidant (21).

The final step of this redox relay where electrons are passed to a terminal electron acceptor is not fully understood. It has been demonstrated using proteins from yeast and of human origin that molecular oxygen can serve as a terminal electron acceptor for disulfide bond formation in solution reconstituted with substrates, PDI and ERO1-Lα (22, 23, 24). This role for molecular oxygen in disulfide bond formation is in line with the demonstration that hypoxia is a strong physiological activator of the UPR in mammalian cells (7, 8, 9), which suggests that proteins misfold in the absence of oxygen. However, this is paradoxical in light of numerous hypoxia-induced ER cargo proteins being secreted under such conditions.

To determine the requirement for oxygen in the ER, we previously directly assayed ER-localized disulfide bond formation in living human cells in the presence and absence of oxygen for two model proteins (8). Isoform 1 of low-density lipoprotein receptor (LDLR) has 30 disulfide bonds concentrated in the N-terminal ligand-binding region (25) (Fig. 1—LDLR) that are formed through nonvectorial folding by distinct cotranslational oxidation and posttranslational isomerization steps (25, 26). Interestingly, we showed that disulfide bonds were readily introduced cotranslationally into LDLR in the absence of oxygen, demonstrating the existence of an alternative terminal oxidant to molecular oxygen (8). Despite proficiency in cotranslational disulfide bond formation, posttranslational disulfide isomerization failed in the absence of oxygen, leading to substantial misfolding and ER retention. This pattern of oxygen dependency for disulfide bond formation and isomerization was recapitulated in another ER cargo protein, influenza hemagglutinin (Flu-HA) (8). This work hence suggested that an oxygen-independent pathway for disulfide bond formation exists, but it cannot fully support productive protein maturation and expression.

**Figure 1:**
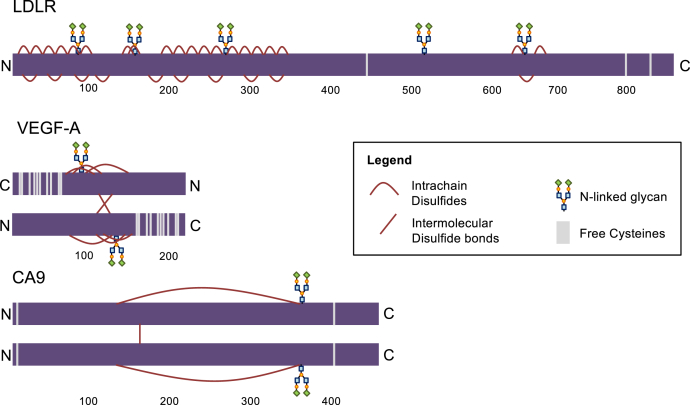
**Overview of glycosylation and disulfide bonds in selected ER cargo.** LDLR contains 30 disulfide bonds concentrated in the N-terminal region, three free cysteines, and five N-linked glycans. VEGF-A is a disulfide linked homodimer containing three intra-chain disulfide bonds surrounding an N-linked glycan. It has a cysteine-rich C terminus. CA9 is a disulfide-linked homodimer containing one N-glycan and two free cysteines.

Here we aimed to determine if the oxygen-independent pathway for disulfide bond formation can fully support the maturation and expression of proteins such as VEGF-A and CA9 that are known to be highly expressed in hypoxia (5, 27). This was determined through radioactive pulse-chase immunoprecipitation studies in which disulfide bond formation and secretion of a highly synchronized newly synthesized protein population could be monitored in the presence and absence of oxygen. Taking a global *in silico* approach, we also compared the number and architecture of disulfides in hypoxia-induced and non-hypoxia-induced proteins to better understand how some proteins may mature independently of oxygen availability. Overall, this study aimed to provide further knowledge on the oxygen dependency of protein expression.

## Results

We sought to determine the requirement for oxygen in the disulfide bond formation of VEGF-A and CA9. VEGF-A_165_ (referred to as VEGF-A in the following) is a disulfide-linked homodimer that contains three intertwined intrachain disulfides comprising a cystine knot structural motif and a cysteine-rich C terminus (28) (Fig. 1—VEGF-A). Formation of the cystine knot is essential for the structural integrity of the monomer as well as the formation of the disulfide-linked dimer (29, 30). CA9 is also a disulfide-linked homodimer (31). It contains one intrachain disulfide as inferred by the proximity of the cysteine residues in the crystal structure (32) and two additional free cysteines (Fig. 1—CA9).

### VEGF-A and CA9 form disulfide bonds independent of oxygen availability

Oxygen dependency of disulfide bond formation and protein maturation was investigated *in cellulo* using a pulse-chase assay (supporting information 1). Cells were exposed to a short pulse of ^35^S-methionine/cysteine in normoxia (21% O_2_) or anoxia (0% O_2_) to create a highly synchronized radioactive pool of newly synthesized protein (33). After removal of the radioactive amino acids, cells were incubated for different periods (“chased”) before they were flooded with an alkylating reagent, N-ethylmaleimide (NEM), to prevent further oxidation of cysteines. Cells were then lysed and proteins of interest were immunoprecipitated and resolved by gel electrophoresis under reducing or nonreducing conditions to interrogate the kinetics of disulfide bond formation.

**Figure 2:**
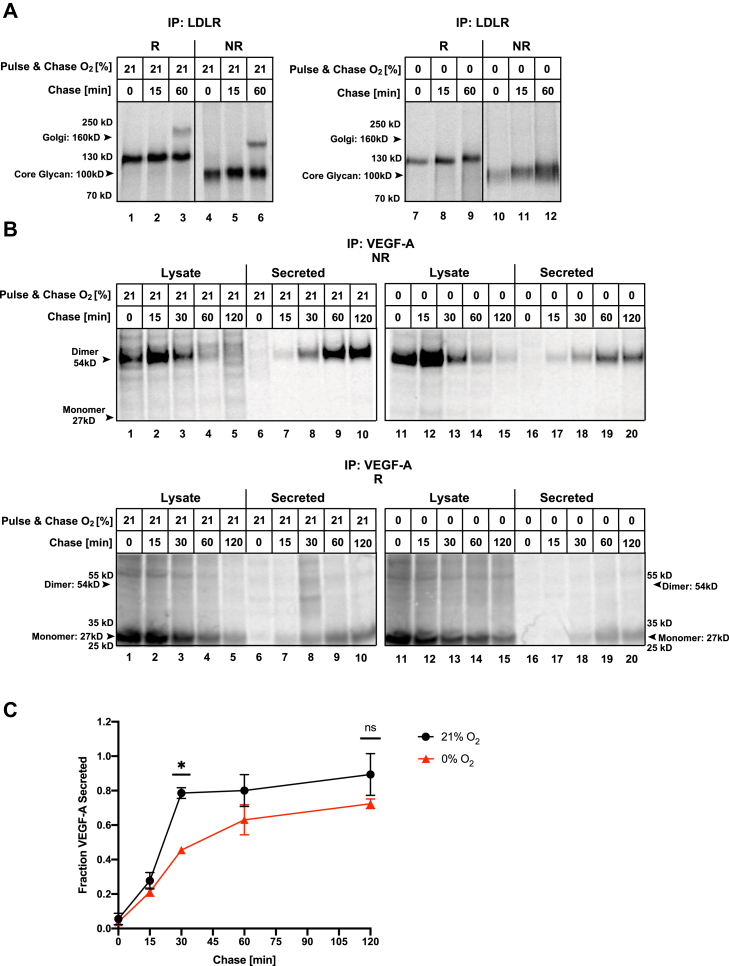
**Oxygen-independent disulfide bond formation in VEGF-A.** HeLa cells transfected with LDLR (*A*) or VEGF (*B* and *C*) were ^35^S pulse labeled under the indicated % O_2_ for 5 min (*A*) or 15 min (*B* and *C*) and chased for the indicated periods. Immunoprecipitated (IP) LDLR (*A*), VEGF-A (*B* and *C*) from lysates or media (secreted) was resolved by SDS-PAGE under reducing (R) or nonreducing (NR) conditions and visualized by autoradiography. *D*, the fraction of VEGF-A secreted was calculated after densitometry of autoradiographs from three independent experiments, expressed relative to the 15-min chase time point. Average ± SEM, n = 3. ∗Statistically significant (*p* < 0.01). ns, nonstatistically significant.

We aimed to compare the oxygen dependency of disulfide bond formation in VEGF-A and CA9 in normoxia and anoxia directly to that of LDLR. As shown previously, LDLR is detected migrating at ∼130 kD under reducing conditions immediately after a 5-min pulse in normoxia (Fig. 2*A*, lane 1), consistent with the fully synthesized protein with its core N-linked glycan. One hour after synthesis, a slower migrating form of LDLR appears, reflecting complex glycosylation in the Golgi (Fig. 2*A*, lane 3) (8, 26). Under nonreducing conditions where disulfide bonds remain present within the protein structure, LDLR migrates more rapidly due to its more compact conformation in comparison to under reduced conditions (Fig. 2*A*, lanes 4–6). This shows that disulfide bonds are introduced during or immediately after translation in LDLR in normoxia. A decrease in overall signal intensity of LDLR is observed in anoxia in comparison to normoxia (Fig. 2*A*, lanes 7–12 *versus* lanes 1–6). This observation is consistent with the reduction in protein synthesis due to rapid PERK-dependent phosphorylation of eIF2α as part of the UPR (7, 34). There was a complete lack of Golgi-localized glycosylation in anoxia (Fig. 2*A*, lane 9 *versus* 3). Similar to normoxia, LDLR migrated faster when it was resolved under nonreducing conditions, reflecting that disulfides are introduced in LDLR during translation also in anoxia (Fig. 2*A*, lane 10 *versus* 7). However, the protein pool becomes more heterogeneous and migrates slower over time (Fig. 2*A*, lanes 10–12), representing a failure to properly isomerize disulfide bonds in anoxia. As such, the defect in Golgi-localized glycosylation in anoxia is likely secondary to impaired disulfide isomerization and an inability to pass ER quality control and traverse to the Golgi.

Unlike LDLR, VEGF-A is a hypoxia-inducible protein that might have evolved to mature in the absence of oxygen. To monitor disulfide bond formation and secretion of VEGF-A, both lysates and media were collected at various time points after the radioactive pulse-chase. Under nonreducing conditions, VEGF-A was detected migrating at ∼54 kD at all time points in both normoxia and anoxia (Fig. 2*B*, lanes 1–20), consistent with the molecular weight of the homodimer. Under reducing conditions, the VEGF-A band was observed at ∼27 kD (Fig. 2*B*), validating that the observed 54kD band indeed represents the disulfide-linked dimer. This observation shows that VEGF-A is rapidly dimerized during or immediately following synthesis in both normoxia and anoxia. It implies that the intramolecular cystine knot forms efficiently in anoxia, since it is known to be required for subsequent dimer formation (29).

We consistently observed an increase in dimer intensity following a 15-min chase time in both oxygen conditions under nonreducing conditions (Fig. 2*B* top, lanes 2 and 12), presumably due to the resolution of undetectable folding intermediates over this time period. VEGF-A then disappeared from the lysate over time (Fig. 2*B*, lanes 1–5 and 11–15), while it appeared in the chase media (Fig. 2*B*, lanes 6–10 and 16–20) as it was secreted. The secretion of VEGF-A was substantial 30 min after synthesis and mostly complete within 120 min in normoxia, (Fig. 2*B*, lanes 1–10) (Fig. 2*C*). In anoxia, the kinetics of VEGF-A secretion over time was slightly slower than in normoxia, but also mostly complete by 120 min after synthesis (Fig. 2, *B* and *C*). Densitometry quantification from multiple experiments supported that VEGF-A is efficiently secreted under both normoxia and anoxia, with a marginal delay in anoxia (*p* = 0.0007) (Fig. 2*C*). On average, about 80% of the total protein fraction was secreted by 120 min after synthesis (Fig. 2*C*). There was no overall loss of signal during the 120-min experimental period, demonstrating that in both normoxia and anoxia, VEGF-A was either secreted or retained in the ER—but not degraded (supporting information 2*A*). Our results show that VEGF-A can successfully complete disulfide bond formation and traverse through the secretory pathway in both normoxic and anoxic conditions.

**Figure 3:**
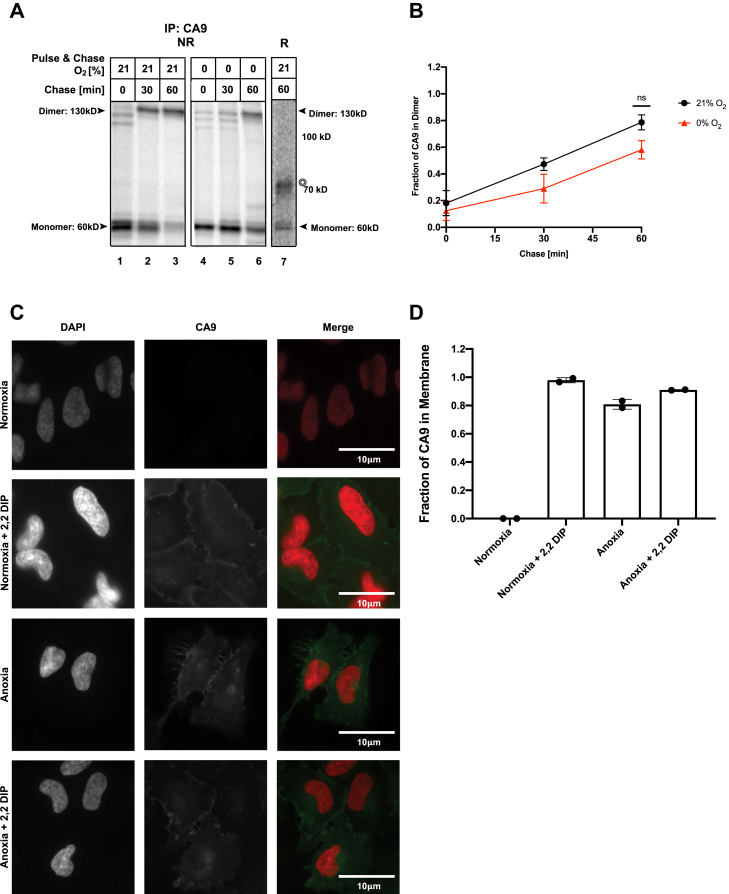
**Oxygen-independent disulfide bond formation in CA9.***A* and *B*, HeLa cells transfected with CA9 were ^35^S pulse labeled under the indicated % O_2_ for 30 min (*A* and *B*) and chased for the indicated periods. Immunoprecipitated (IP) CA9 (*A*) from lysates was resolved by SDS-PAGE under reducing (R) or nonreducing (NR) conditions and visualized by autoradiography. ◎ denotes a nonspecific band (see supporting information 3). *B*, the fraction of CA9 dimer was calculated after densitometry of autoradiographs from three independent experiments, expressed relative to the 0-min chase time point. Average ± SEM, n = 3. *C* and *D*, HeLa cells were exposed to normoxia or anoxia (0% O_2_) with and without 100 μM 2,2 DIP for 24 h before fixation and immunofluorescence staining for CA9 and nuclei (DAPI). *D*, fraction of CA9 present on the membrane (average ± SD., n = 2). ns, nonstatistically significant.

To test the oxygen dependency of disulfide bond formation in a hypoxia-induced membrane-bound ER cargo, CA9 was probed. After synthesis in normoxia or anoxia, CA9 migrated mostly at ∼55 kD, consistent with the monomeric form (Fig. 3*A*, lanes 1 and 4, supporting information 3). Progressive dimer formation could be seen in normoxia between 30 and 60 min after CA9 synthesis. (Fig. 3*A*, lanes 2 and 3). Sample reduction confirmed that the higher-molecular-weight band represents the disulfide-linked dimer (Fig. 3*A*, lane 7). In anoxia, dimer formation was delayed in comparison to normoxia (Fig. 3*A*, lane 2 *versus* 5), but a substantial amount of the CA9 protein pool reached a dimer state by 1 h after synthesis (Fig. 3*A*, lanes 3 and 6). Densitometry quantification from multiple experiments showed that by 60 min after synthesis, 80% of the total protein pool was detected as dimers in normoxia (Fig. 3*B*). Approximately 50% of the protein population formed dimers over the same time period in anoxia. Although this suggests a delay in CA9 dimerization in anoxia, this difference was not statistically significant (*p* = 0.4). Densitometry quantification confirmed that there was no overall loss of signal during the 60-min experimental period suggesting that CA9 was neither degraded nor aggregated (supporting information 2*B*).

To validate that dimerized CA9 could pass ER quality control and traverse the secretory pathway to reach its final plasma membrane destination under anoxia, we quantified CA9 localization by immunofluorescence (IF) (Fig. 3*C*). We induced endogenous CA9 expression by hypoxia and/or 2,2 dipyridyl (2,2-DIP), which activate the transcription factor HIF. Both treatments resulted in similar upregulation of CA9 mRNA and protein (supporting information 4). Under all conditions, 80% of CA9 was detected in the plasma membrane (Fig. 3*D*), demonstrating that although dimerization of CA9 may be somewhat delayed in anoxia, the protein proceeds through the secretory pathway to be expressed at the cell surface in the absence of oxygen.

Taken together, the data in Figures 2 and 3 demonstrate the existence of a diverse spectrum of intrinsic abilities of different proteins to form the required disulfide bonds and progress through the secretory pathway in the absence of oxygen.

### The maturation of LDLR does not improve with prolonged hypoxia exposure

Our data suggest that a subset of proteins, exemplified by LDLR, cannot mature fully in the absence of oxygen and are the source of ER stress and UPR in hypoxia. The UPR is an adaptive response that promotes hypoxia tolerance by inhibiting protein synthesis (preventing further stress) and transcriptionally upregulating proteins involved in autophagy and redox homeostasis (10, 11, 12, 35). It is also frequently assumed that UPR-dependent transcriptional upregulation of ER folding factors can increase the folding capacity of the ER and hence mitigate ER stress. To test this idea, we sought to determine if pre-exposing cells to anoxia would aid in the maturation process of LDLR through the recruitment of folding factors induced by the UPR.

**Figure 4:**
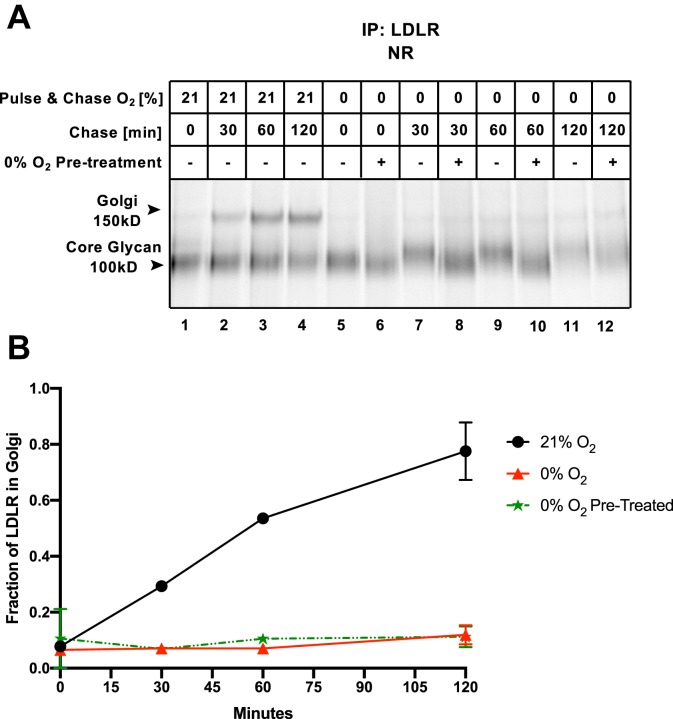
**Preconditioning with anoxia does not increase the folding proficiency of LDLR**. HeLa cells were seeded and transfected with LDLR the next day. Twenty-four hours thereafter, cells were exposed to 0% O_2_ for 18 h (“0% O_2_ Pre-treatment”), followed by ^35^S pulse labeling for 30 min under the indicated % O_2_. Following the indicated chase times, LDLR was immunoprecipitated (IP) from total cell lysates and resolved by nonreducing (NR) SDS-PAGE (*A*). *B*, the fraction of LDLR in Golgi was calculated after densitometry of autoradiography (average ± SD, n = 2).

Interestingly, we found that pre-exposing cells to hypoxia did affect the folding fate of LDLR, but not in a productive manner. As observed before, LDLR did not undergo Golgi-localized glycosylation during acute hypoxia, in comparison to normoxia (Fig. 4*A*, lanes 1–3 *versus* 7, 9, 11), and pretreatment with 18 h of anoxia did not change that (Fig. 4*A*, lanes 8, 10, 12). Densitometric quantification confirmed that whereas close to 80% of the synthesized LDLR existed in the Golgi-modified form after 120 min in normoxia, a mere 10% carried this modification when synthesized and matured in either acute (1 h) or chronic (18 h) anoxia (Fig. 4*B*). However, rather than migrating slower overtime under anoxic conditions (Fig. 4*A*, lanes 5, 7, 9 and 11), LDLR remained mainly in the immature oxidized form that is present immediately after the synthesis when cells were pre-exposed to anoxia (Fig. 4*A*, lanes 6, 8, 10, and 12). Thus, although chronic hypoxia alters the chemical or physical composition of the ER in ways that affect protein folding, we observed no evidence for increased folding capacity that would lead to improved protein expression.

In summary, these experiments show that long-term hypoxia can alter the ER folding micromilieu to affect disulfide bond processing. However, we found no evidence to support the idea that prolonged hypoxia would lead to adaptive responses and improve folding capacity.

### Disulfide bond formation of ER cargo is disrupted after a DTT challenge in hypoxia

**Figure 5:**
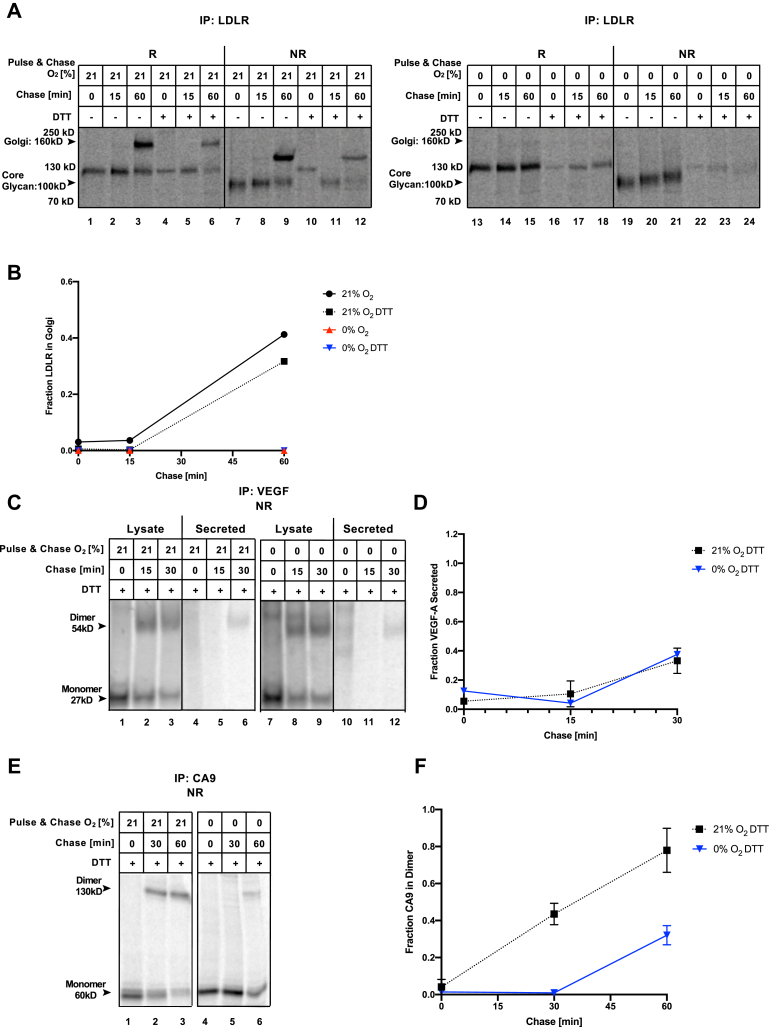
**Delayed oxygen-independent disulfide bond formation after translation in DTT.** HeLa cells transfected with LDLR (*A* and *B*), VEGF-A (*C* and *D*), or CA9 (*E* and *F*) were ^35^S pulse labeled in the presence of 5 mM DTT for 15 min under the indicated % O_2_. Proteins were subsequently allowed to fold in DTT-free media for the indicated chase times. The indicated immunoprecipitated (IP) proteins were resolved under reducing (R) or nonreducing (NR) conditions by SDS-PAGE and visualized by autoradiography. Fraction of LDLR in Golgi (*B*), VEGF secreted (*D*), or CA9 dimer (*F*) was quantified from autoradiographs, relative to the 0-min chase (LDLR and CA9) or 15 min chase (VEGF) time point. *B*, n = 1. *D* and *F*, average ± SEM, n = 3.

Here (Fig. 2*A*) and in previous work (8), we have shown that cotranslational LDLR disulfide oxidation is an oxygen-independent process, while posttranslational disulfide isomerization is oxygen-dependent. This observation was unexpected, given that the isomerization process is electron neutral and, therefore, in principle, not dependent on a terminal electron acceptor. We wondered if the competency for initial oxidation in anoxia was functionally linked to translation or an intrinsic property of the protein. We therefore uncoupled initial oxidation from translation by ^35^S radioisotope labeling in the presence of dithiothreitol (DTT), which keeps LDLR in a fully reduced form (Fig. 5*A*, lanes 4 and 10). We then removed DTT to allow the proteins to form disulfide bonds posttranslationally. In the presence of oxygen, LDLR was oxidized to form the faster migrating compact conformation within 15 min after release from DTT (Fig. 5*A*, lane 11) and progressed to undergo Golgi-localized glycosylation with similar kinetics as observed when the protein was translated in the absence of DTT (Fig. 5*A*, lane 6 *versus* 3) (Fig. 5*B*). The presence of DTT in combination with hypoxia exposure caused reduced signal intensity due to the inhibition of protein synthesis (Fig. 5*A*, lanes 16–18 and 22–24). Nevertheless, it appeared that most of LDLR remained reduced when released from DTT in the absence of oxygen, with some very modest oxidation over time represented by a smearing of the band (Fig. 5*A*, lanes 22–24) (Fig. 5*B*). This suggests that the ability of LDLR to undergo initial oxidation in the absence of oxygen is tightly coupled to translation and cannot efficiently be postponed to the posttranslational folding phase.

Based on these observations, we then sought to determine if oxidation of VEGF-A is also tightly coupled to translation. In previous experiments (Fig. 2*B*), we had only detected trace of a single monomer band of VEGF-A, since dimerization occurred rapidly during/after synthesis. When VEGF-A was translated in the presence of DTT, a migrating monomer band was present (Fig. 5*C*, lanes 1–3 and 7–9), representing the fully reduced protein devoid of intrachain disulfide bonds. VEGF-A could mature after release from DTT in normoxia and anoxia to be secreted as a disulfide-linked dimer (Fig. 5*C*, lanes 6 and 12). However, its monomer oxidation, dimer formation, and secretion were delayed after release from DTT in both oxygenation conditions (Fig. 5*C*, lanes 6 and 12, compared with Fig. 2*D*) (Fig. 5*D*). We also uncoupled CA9 oxidative folding from translation by the same approach. Disulfide-linked dimers were made with somewhat slower kinetics in normoxia after the protein was translated in the presence of DTT. However, in anoxia, dimerization of CA9 was severely affected (Fig. 5*E* compared with Fig. 3*A*) (Fig. 5*F*).

Taken together, these experiments show that in the presence of oxygen, most ER cargo can mature if disulfide bond formation is postponed to the posttranslational phase, albeit with slower kinetics. However, disulfide bond formation can represent a more substantial challenge when postponed to after translation in anoxia. This suggests that the competency for forming correct disulfides in hypoxia is not an intrinsic property of the protein, but rather dependent on the context in which it folds.

### Hypoxia-induced proteins have different disulfide bond architecture compared with other ER cargo

**Figure 6:**
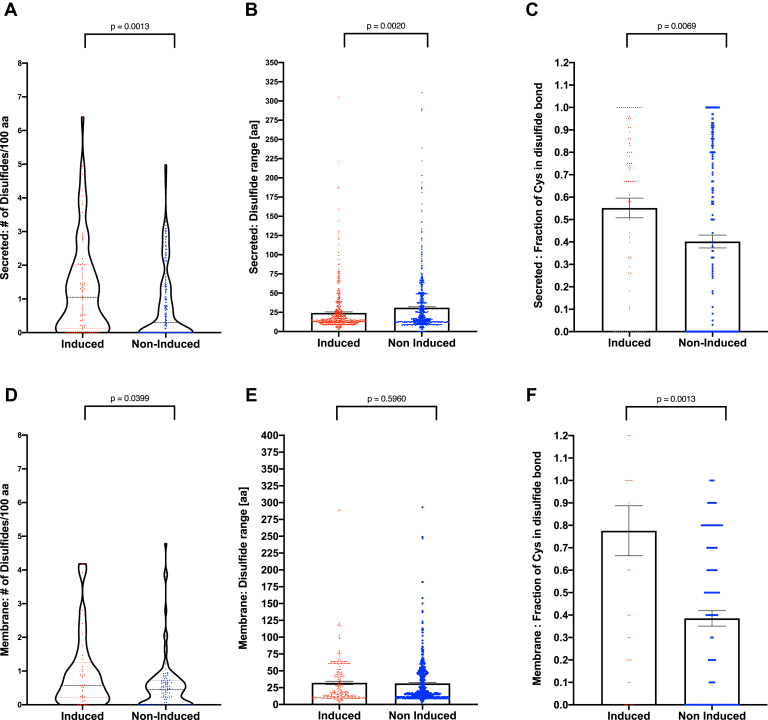
**Global analysis of disulfide bonds in ER cargo.** The analysis pipeline can be found in supporting information 5. Each individual ER cargo protein is presented as a single point across all figures. Analysis has been performed in two separate ER cargo protein groups of hypoxia-induced and non-hypoxia-induced proteins. Secreted proteins (*A–C*) and membrane proteins (*D–F*). The number of disulfide bonds per 100 amino acids is illustrated in a violin plot with median value and interquartile range indicated (*A* and *D*). The range of disulfide bonds expressed as the number of amino acids between the paired cysteines (*B* and *E*) and the fraction of cysteines participating in an annotated disulfide bond (*C* and *F*) are illustrated with bar graphs. Bars represent the mean values with SEM indicated.

In combination, work presented here and elsewhere (8) on the maturation of four ER cargo (LDLR, Flu-HA, CA9, and VEGF) *in cellulo* in the presence and absence of oxygen reveals that the ability of a protein to utilize an oxygen-independent pathway for disulfide bond processing varies widely. We wondered if the superior ability of VEGF-A and CA9 to fold in hypoxia compared with LDLR and Flu-HA (8) could be the result of an evolutionary selection pressure for hypoxia-induced proteins to be successfully matured in the absence of oxygen. We reasoned that such a selection pressure might be reflected in traits such as disulfide bond abundance or architecture. To address this, we used previously obtained microarray data (8) from two cell lines to establish two distinct groups of ER cargo; proteins whose transcripts were induced twofold or more by hypoxia (n = 112), and proteins whose transcripts were not induced by hypoxia (n = 313) (supporting information 5). We also separated secreted from membrane-bound proteins. We then analyzed the abundance and architecture of disulfide bonds (n = 2124) as annotated by Uniprot (www.uniprot.org) in these proteins (Fig. 6).

Interestingly, in secreted proteins, we found that hypoxia-induced proteins had a slightly higher number of disulfide bonds than noninduced proteins (1.3 ± 0.16 SEM disulfide bonds per 100 amino acids *versus* 0.83 ± 0.08 SEM, *p* = 0.0013) (Fig. 6*A*). This difference was recapitulated when we analyzed membrane proteins (0.95 ± 0.19 SEM disulfide bonds per 100 amino acids *versus* 0.58 ± 0.08 SEM, *p* = 0.04) (Fig. 6*D*). This difference suggests that there has been no selection pressure against disulfide bonds in hypoxia-induced proteins in general. Given our data demonstrating the inability of LDLR to complete disulfide isomerization without oxygen, we sought to determine if there might have been a selection pressure against a requirement for isomerization in hypoxia-induced proteins. We reasoned that a need for isomerization might be associated with the range of disulfide bonds (*i.e.*, the distance between the paired cysteines) and the presence of cysteines not participating in disulfide bonds. In long-range disulfide bonds, the cysteine translated first has a long lifetime as a free thiol with the opportunity to react with other cysteines in its proximity resulting in a non-bonafide disulfide bond that needs to be resolved. Similarly, unpaired free cysteines can erroneously react with cysteines intended for other partners. We observed a broad distribution of ranges of disulfide bonds in both protein groups (Fig. 6, *B* and *E*). However, in secreted proteins, in line with the hypothesis, the average range of a disulfide bond in hypoxia-induced ER cargo was shorter than in noninduced proteins (24.25 amino acids ±1.3 SEM *versus* 31.02 ± 1.2 SEM, *p* =0.0020) (Fig. 6*B*). In this parameter, there was no difference between the groups of membrane-bound proteins (Fig. 6*E*). However, for both secreted and membrane-bound proteins, a higher fraction of cysteines participate in a disulfide bond in hypoxia-induced proteins, compared with noninduced proteins (*p* = 0.0069, 0.0013) (Fig. 6, *C* and *F*). Consequently, hypoxia-induced proteins contain fewer free cysteines (*i.e.*, cysteines that do not participate in a disulfide bond) than proteins not induced by hypoxia.

These data demonstrate that despite broad distributions across the groups, statistically significant differences exist in both the frequency and architecture of disulfide bonds between proteins induced by hypoxia and other proteins. These differences may reflect the evolution of hypoxia-induced proteins to rely less on oxygen for productive protein folding.

## Discussion

**Figure 7:**
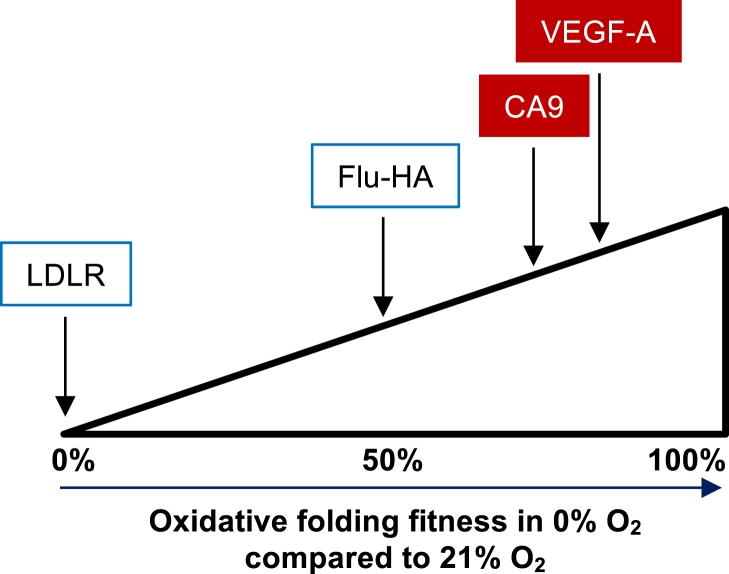
Illustration of oxidative folding fitness in hypoxia for four ER cargo. Oxidative folding fitness in 0% O_2_ was assigned as the percentage of the protein that achieves a conformation similar to the one in normoxia for the longest time point experimentally assessed. Data for LDLR and Flu-HA are from Fig. 2*A* and Koritzinsky et al. (8). Data for CA9 and VEGF-A are from Figures 3*B* and 2*C* respectively. Proteins represented by a red box (VEGF-A and CA9) are transcriptionally induced by hypoxia. Folding fitness in hypoxia varies widely between different proteins, and hypoxia-induced proteins tend to fold better in the absence of oxygen.

In our previous work and here, we have for the first time characterized the oxidative folding capacity of four ER cargo proteins (LDLR, Flu-HA, VEGF-A, CA9) in the absence of oxygen *in cellulo*. These ER cargo proteins have shown a remarkable distribution in their intrinsic ability to achieve the correct disulfide bond conformation in anoxia. If we take a bird's eye view, we can define “oxidative folding fitness” as the overall ability of a protein for disulfide bond processing, passing ER quality control, and traversing through the secretory pathway. We have identified a broad range of oxidative folding fitness in anoxia within the four proteins that we have probed (Fig. 7). In spite of being competent in initial oxidation, LDLR has poor oxidative folding fitness in anoxia since it does not achieve its native fold and does not pass quality control to leave the ER under such conditions (Fig. 2*A*) and (8). Flu-HA has intermediate oxidative folding fitness in anoxia, with approximately 50% of the protein pool misfolding in the absence of oxygen (8). CA9 and VEGF-A have high oxidative folding fitness in the absence of oxygen, as both proteins reach their disulfide-linked dimer state with only minor delay in anoxia compared with normoxia (74% in Fig. 3*B* and 81% in Fig. 2*C* respectively). Based on these observations, we can position LDLR, Flu-HA, CA9, and VEGF-A at 0%, 50%, 74%, and 81% folding fitness in anoxia compared with normoxia (Fig. 7).

The broad range in oxidative folding fitness in anoxia across various proteins implies that the process of disulfide bond formation and isomerization can be a vital contributor to differential protein expression in the extracellular space when oxygen is restricted. From the four proteins that we have probed, an interesting pattern emerged. Hypoxia-induced proteins such as VEGF-A and CA9 have a higher oxidative folding fitness in the absence of oxygen than proteins such as LDLR and Flu-HA, which have no well-described specific roles in hypoxia (Fig. 7). Certainly, the investment of cellular energy in the production of proteins such as VEGF-A and CA9 in hypoxia would be wasteful if the proteins could not proceed to fold, and their expression is of great importance to the organism under oxygen-restricted conditions. Selective oxidative folding in hypoxia and anoxia may represent an opportunity for the organism to prioritize expression of protein of particular importance under such conditions.

As such, we speculate that hypoxia-induced proteins have been subject to a selection pressure and evolved with an ability to utilize the oxygen-independent pathway(s) for oxidative protein folding. Our global *in silico* analysis of disulfide bond organization in ER cargo supports this idea. Our analysis is limited by the accuracy and completion of the database, as we assumed that all cysteines not annotated to participate in a disulfide bond formation are free thiols. However, we have no reason to believe that there are systematic biases between the analyzed groups in this respect. Although the traits determining the ability of a protein to utilize an oxygen-independent folding pathway are likely highly complex, the existence of statistically significant differences in the number of free cysteines and the architecture of disulfide bonds between hypoxia-induced and other proteins suggest that these traits may contribute to oxidative folding fitness in anoxia.

What mechanistically underlies the oxidative folding fitness in hypoxia remains unknown. The folding fitness of a protein could be related intrinsically to the nature of each disulfide bond, to the folding factors on which they are dependent, and/or the availability of an alternative terminal electron acceptor to oxygen. In addition, some proteins may have higher tolerance for errors and as such rely less on native disulfide bonds to pass quality control. Although this explanation could be true for some proteins, it is unlikely to apply to proteins such as VEGF-A since mutation of cysteines in the cystine knot results in poor dimer formation (29). It is also possible that proteins with few disulfides or a “simple” disulfide architecture may have a folding advantage in hypoxia. Several of our experimental observations are consistent with a need for disulfide isomerization being a deterrent of oxidative folding fitness. Also, hypoxia-induced proteins contain more but shorter ranged disulfide bonds as well as fewer free cysteines than other proteins, which is in line with disulfide isomerization, *not* oxidation, being limiting.

A requirement for oxygen in disulfide isomerization may be surprising, given that a terminal oxidant is in principle not needed in the isomerization process. However, although PDI can independently rearrange disulfide bonds in client proteins, reduction and oxidation have been shown to be decoupled in some proteins (36). For LDLR, the PDI family member ERdj5 serves as a reductase in the isomerization process, followed by an unknown oxidase for the subsequent oxidation. Taken together, these data suggest that general protein oxidation can use other terminal electron acceptors than oxygen, but that the oxidase that follows ERdj5-dependent reduction is oxygen-dependent. As such, we propose that CA9 and VEGF-A are proficient folders in anoxia because of their lack of requirement for disulfide isomerization. We speculate that the thermodynamic cotranslational folding of these two proteins leaves less probability for the wrong cysteines to encounter during the process.

Our finding of no requirement for oxygen in initial disulfide oxidation is also surprising given that the only known physiologically relevant terminal electron acceptor for the canonical PDI-ERO1 redox cascade is molecular oxygen (22, 24). Interestingly, VEGF-A is thought to use this canonical pathway, since knocking down ERO1-Lα in normoxia reduced VEGF-A secretion (37). If the VEGF-A folding pathway involves ERO1-Lα in both normoxia and anoxia, this implies that ERO1-Lα can utilize alternative electron acceptors to oxygen for its reoxidation. This is in line with the observation that anoxic yeast cells are sensitive to losing functional ERO1p expression (21), suggesting that ERO1p retains functionality in the absence of oxygen. For PDI, studies have indicated that PDI family members have varying levels of hypoxia inducibility, suggesting that some family members may have unique roles in hypoxia (38).

Our study also demonstrated that hypoxia preconditioning changed the folding fate of LDLR in anoxia but did not suggest that this change was productive (Fig. 4, *A* and *B*). This was counter to an expectation that hypoxia would confer adaptation and that activation of HIF and the UPR might increase the abundance of limiting folding factors. It is still possible that upregulated folding factors may be selective to a certain subpopulation of ER cargo and aid their folding exclusively. It is also possible that increased abundance of folding factors is useful when the amounts of oxygen present are limited (hypoxia) rather than absent (anoxia).

In summary, our findings demonstrate the existence of a vast range in the ability of different proteins to achieve native disulfide bonds in the absence of oxygen. It suggests that hypoxia-induced proteins have a superior oxidative folding fitness in hypoxia. This may be supported by specific traits in disulfide architecture and underlies their high functional expression during oxygen deprivation. Learning more about the mechanisms responsible for the expression of hypoxia-inducible proteins such as VEGF-A and CA9 can further our understanding of (patho)physiological conditions such as development, ischemia, and the human tumor microenvironment.

## Experimental procedures

### Cell culture and hypoxia

HeLa cells (cervix carcinoma, ATCC: CCL-2) were maintained in Dulbecco's modified Eagle's medium (Invitrogen) supplemented with 10% fetal bovine serum (FBS) (Gibco) and 1% Amphotericin B (Gibco) and Penicillin-Streptomycin (Invitrogen). Cells were grown adherently at 80% confluency on glass dishes that, in contrast to plastic, do not release oxygen. Cells were exposed to anoxia (0.0% O_2_) in a H85 HypOxystation (Don Whitley Scientific). Experiments were conducted after inserting the cells into the hypoxic chamber for 1 h. All media and solutions were equilibrated to the desired oxygen concentrations before use.

### Transfections

Plasmids included pcDNA3-LDLR encoding the full-length human low-density lipoprotein receptor (26) and pCMV_XL5-CA9 encoding full-length human carbonic anhydrase 9 (Origene). The coding sequence of VEGF-A_165_ was amplified from HeLa cell cDNA using the primers 5′-caccatgaactttctgctgtcttgggtg-3′ and 5′-tcaccgcctcggcttgtcacatctgcaagtacgttcgtt-3′, blunt-end cloned into the Gateway pENTR (Life Technologies Inc), and subcloned to produce sequence-verified pCMV6-VEGF-myc-FLAG. Cells were transfected using Lipofectamine 2000 according to manufacturer's protocol.

### Pulse-chase assay

Cells were rinsed with phosphate-buffered saline (PBS) and starved of methionine and cysteine for 30 min. Newly synthesized proteins were radioactively labeled for 5–30 min using 50 μCi EasyTag EXPRESS 35S-Protein Labeling Mix (PerkinElmer) per 4-cm dish. Labeling times were optimized for each protein of interest, using the shortest pulse time that produced adequate signal. Incorporation of radioactive amino acids was stopped by adding chase media (DMEM with 10% FBS, 20 mM HEPES, 5 mM cysteine, 5 mM methionine, 1 mM cycloheximide). At the end of the chase period, cells were flooded with ice-cold PBS containing 20 mM NEM to alkylate free cysteines to prevent further oxidations. Anoxia samples were flooded with PBS, including NEM in the anoxia chamber. When specified, cotranslational disulfide bond formation was prevented by including 5 mM DTT in the labeling media. Cells were then washed three times and incubated in DTT-free chase media. Cells were lysed in 20 mM NEM-containing Triton lysis buffer (20 mM MES, 100 mM NaCl, 30 mM Tris-HCl, pH 7.4, 0.5% Triton X-100, 60 mM N-octylglucoside, and 1 mM EDTA) for LDLR or RIPA buffer (150 mM NaCl, 1% NP-40, 0.5% Na-deoxycholate, 0.1% SDS, and 50 mM Tris-HCl, pH 7.5) with 1% benzonase nuclease (Sigma). RIPA and Triton lysis buffer for VEGF-A and CA9 both included a 1× Halt protease inhibitor cocktail (Thermo Scientific).

### Immunoprecipitation

In total, 40 μl of Protein A or G Dynabeads (Invitrogen) was incubated with either 5 μl anti-LDLR (rabbit polyclonal antiserum (26)) or 8 μl anti-CA9 (R&D systems) for 2 h at room temperature with rotation. For the detection of VEGF-A, 30 μl of Protein G was incubated with 4 μl of anti-FLAG (Cell Signaling Technologies) overnight at 4 °C with rotation. Following antibody incubation on beads, protein lysates were then incubated overnight at 4 °C with rotation. Beads were then washed five times in TBST at room temperature and were resuspended and boiled in elution buffer (1 M Tris-HCl, pH: 7.5, 4% SDS). The samples were resolved on SDS-PAGE gels with or without reduction with 2-mercaptoethanol (β-ME). Gels were stained with Coomassie Brilliant Blue (Sigma), fixed (30% methanol, 10% acetic acid), and neutralized (30% methanol in PBS) before drying and exposing to a storage phosphor screen (GE Healthcare). Signals were detected on a variable mode imager (Typhoon 9410; GE Healthcare). A vertical solid line in the figures denotes that the autoradiograph was cropped after running samples on the same gel. A gap between autoradiographs signifies that samples were run on different gels.

### Western blot

Cells were washed twice with PBS, then lysed using RIPA buffer (50 mM, Tris-HCl: pH 7.4, 1% NP-40, 0.25% Na-deoxycholate, 150 mM NaCl, 1 mM EDTA), supplemented with protease inhibitor (Thermo Fisher Scientific). Protein concentration was determined by Bicinchoninic Acid (BCA) Protein Assay kit (Thermo fisher Scientific) and 30 μg protein prepared with Bolt lithium dodecyl sulphate sample buffer (Thermo Fisher Scientific) with or without 1 mM DTT. Proteins were resolved on Bolt 4 to 12% Bis-Tris Plus Gels and transferred to a polyvinylidene difluoride membrane (immobilon-P, Millipore). Membranes were incubated in Licor blocking buffer and decorated with the following primary antibodies: Anti-Flag (1:200, 14793S, Cell Signaling Technology), Anti-CA9 (1:1000, Gift from S. Pastoreková), Anti-LDLR (1:2000, PAB15520, Abnova), Anti-HIF-1α (1:1000, 610958, BD Bioscience), and Anti-Vinculin (1:3000, ab129002, Abcam). Secondary antibodies included IRDye 680RD and/or IRDye 800CW (1:10,000, Licor). Proteins were visualized using Licor Odyssey CLx Imaging System (Licor). Western blots showing the molecular weight of monomers and dimers of each protein are shown in supporting information 6.

### Immunofluorescence and image analysis

Cells were seeded on coverslips and exposed to anoxia (0.0% O_2_) and/or 100 μM of 2,2 DIP for 24 h. Cells were fixed in ice-cold 100% methanol for 10 min and incubated in blocking solution (0.02% Tween20, 1X PBS, 3% BSA) for 1 h. Cells were incubated with Anti-CA9 (1:200, Gift from S. Pastoreková) overnight at 4 °C and incubated with Alexa Fluor 568 goat anti-mouse IgG (1:500, Invitrogen) for 1 h at room temperature. Cells were then washed three times before mounting the coverslips on Vectashield containing DAPI. Preparations were imaged at 60× magnificent with the resolution of 0.1083 μm/pixel using a Nikon fluorescence microscope.

A pseudo-watershed cell segmentation was interactively applied in QuPath (39). After selecting background regions, a 2 μm dilation was applied to stimulate the area of a cell membrane. Inverse of the background annotation was taken and classified as the whole cell area. Mean CA9 intensity was measured in the whole cell and cell membrane areas. Localization of CA9 to the membrane was defined as the ratio of CA9 intensity within the membrane area to CA9 intensity within the whole cell area.

### RT-qPCR

RNA was extracted using the RNeasy Mini Kit according to manufacturer's protocol (Qiagen). Quantity and quality of the RNA were determined using the Nanodrop (Thermo Fisher Scientific). RNA (0.5 μg) was reverse transcribed using qScript cDNA supermix (Quantabio) and qPCR was performed using SensiMix SYBR and Eppendorf Mastercycle ep Gradient S PCR system. HPRT1 was used as a housekeeping control gene for normalization. All cDNA samples were measured in triplicate using the following primers: CA9 (F): GTGCCTATGAGCAGTTGCTGTC; CA9 (R): AAGTAGCGGCTGAAGTCAGAGG; HPRT1 (F): CCTGGCGTCGTGATTAGTGAT; HPRT1 (R): AGACGTTCAGTCCTGTCCATA.

### *In silico* disulfide analysis

To identify ER cargo, all human genes in UniProt were filtered for the presence of a signal peptide (Sequence annotation: “Signal”); subcellular location including “secreted” or “cell membrane”; and subcellular locations excluding “endoplasmic reticulum” or “Golgi apparatus.” The resulting Gene IDs were cross-referenced with a pool of genes that were induced (>2 fold) or suppressed/noninduced (<1.2 fold) following 24 h anoxia in HCT116 and/or HepG2 cells (8). The number of disulfide bonds, the number of disulfide bonds per 100 amino acids, and the distribution of disulfide bond lengths (extracted from Uniprot: http://uniprot.com) were calculated for each ER cargo protein. The fraction of cysteines involved in disulfide bonds were also calculated for each protein as (2D +1I)/total # cysteines, where D= # intrachain disulfides and I = # of interchain disulfides. Intracellular (cytosolic) portions of the membrane bound proteins were excluded from the analysis.

### Statistical analysis

Statistical significance after densitometry quantification was determined *via* Student's *t*-test. The *in silico* data set was analyzed using the Mann–Whitney test.

## Data availability

All data are contained within the article.

## Supporting information

This article contains supporting information (8).

## Conflict of interest

The authors declare no conflicts of interest with regard to this article. M.K. has holdings in Northern Biologics and has a personal associate who is consultant for Versant Ventures and is Corporate Board Director/Trustee and has holdings in Northern Biologics. These associations do not relate to the work presented here.
