# Supplementary material for: Oxygen-independent disulfide bond formation in VEGF-A and CA9
Source: J Biol Chem. 2021 Mar 3;296:100505. doi: 10.1016/j.jbc.2021.100505 (PMC8065220; doi:10.1016/j.jbc.2021.100505)
Supplement: Supporting information 1 to 5 [file mmc1.pdf]

Oxygen-independent disulfide bond formation in VEGF-A and CA9
Supporting Information

Fiana Levitin[1¶], Sandy Che-Eun S. Lee[1-2¶], Stephanie Hulme[1-2], Ryan A. Rumantir[1-2], Amy S. Wong[1-2], Marmendia R. Meester[1-2], Marianne Koritzinsky[1-4§]

[1] Princess Margaret Cancer Center, University Health Network, Toronto, Canada

[2] Institute of Medical Science, University of Toronto, Toronto, Canada

[3] Department of Radiation Oncology, University of Toronto, Toronto, Canada

[4] Department of Medical Biophysics, University of Toronto, Toronto, Canada

[¶] These authors contributed equally to this work
[§] Corresponding Author: Dr. Marianne Koritzinsky (MK)

101 College Street, Room 12-314
Princess Margaret Cancer Research Tower
Toronto, Ontario, Canada
M5G 1L7
Email: Marianne.Koritzinsky@uhnresearch.ca
Phone: +1 416 581 7841

Running title: Disulfide bond formation in hypoxia

Keywords: Carbonic anhydrase 9 (CA9), vascular endothelial growth factor (VEGF), low-density lipoprotein receptor (LDLR), protein folding, disulfide, glycosylation, endoplasmic reticulum (ER), hypoxia, tumor microenvironment

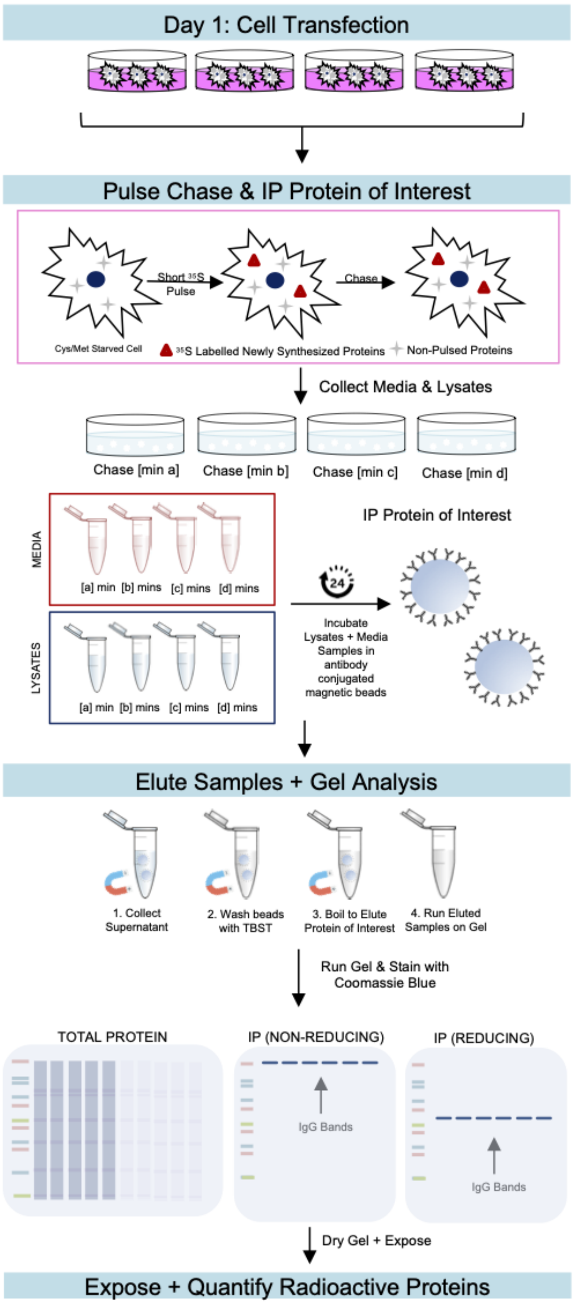

Supporting Information 1. Experimental overview. Cells were transfected and pulse labelled with ^{35}S-methionine/cysteine. Media and lysates were collected after variable chase times and immunoprecipitated with an antibody-conjugated magnetic bead. Eluted samples were then run on a gel with or without a reducing reagent. Total proteins were run as an experimental control for labelling efficacy. Gels were stained with coomassie blue to visualize the protein content (mainly IgG), verifying the retention or disruption of disulfide bonds post lysis. Then gels were dried and exposed on a phosphor screen over days/weeks to visualize radioactive protein.

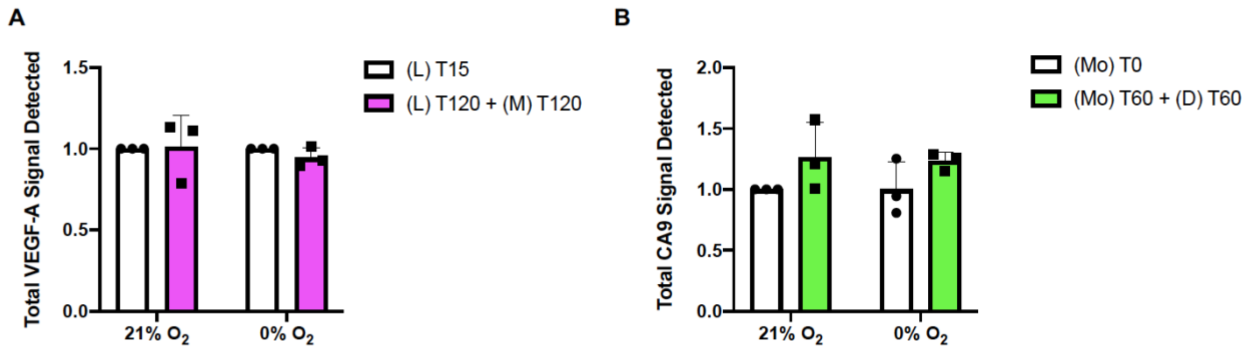

Supporting Information 2. VEGF-A and CA9 are not degraded in anoxia. HeLa transfected with VEGF-A and CA9 were ^{35}S pulse labelled for 15 (A) and 5 minutes (B) under the indicated % O_2 and chased for the indicated time periods. Immunoprecipitated VEGF and CA9 from total lysate (L) and media (M) was resolved by SDS-PAGE under non-reducing conditions. T = chase time in minutes. Mo = monomer detected in total lysate. D = dimer detected in total lysate. Total VEGF-A dimer and CA9 dimer and monomer signal detected was calculated after the densitometry of autoradiography from three independent experiments. Average ± SD., n = 3.

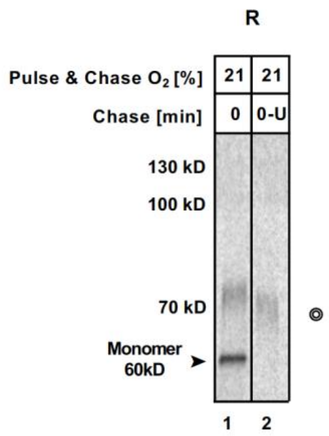

Supporting Information 3. CA9 antibody produces non-specific band under reducing conditions. HeLa cells were seeded and transfected with CA9 the next day. 24 hours thereafter, cells were ^{35}S pulse labelled for 5 min under 21% O_2 in pulse media and was collected right after the pulse. Immunoprecipitated CA9 from lysates in the CA9 transfected and CA9 Untransfected (0-U) was resolved by SDS-PAGE under reducing (R) conditions. ◎ denotes a non-specific band.

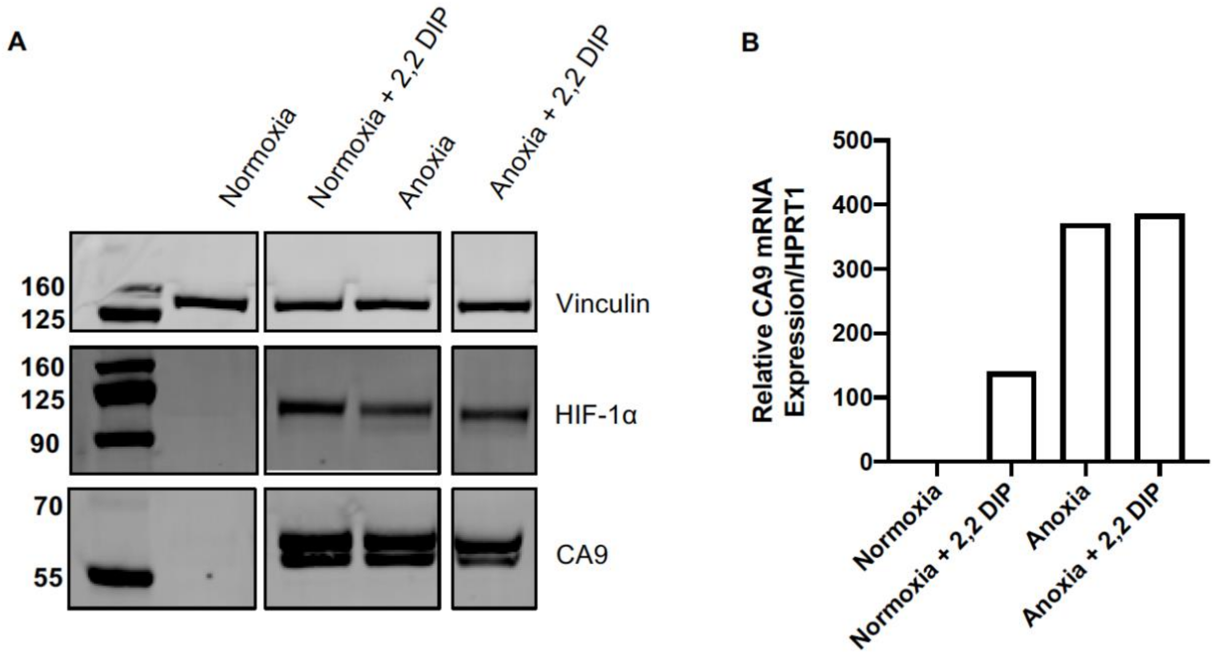

Supporting Information 4. CA9 is induced by hypoxia and 2,2 DIP. HeLa cells were exposed to 0% O_2 and/or 100μM 2,2 DIP for 24 hours. (**A)** Western blotting for the indicated proteins. (**B)** CA9 mRNA expression relative to HPRT1 normalized to Normoxia.

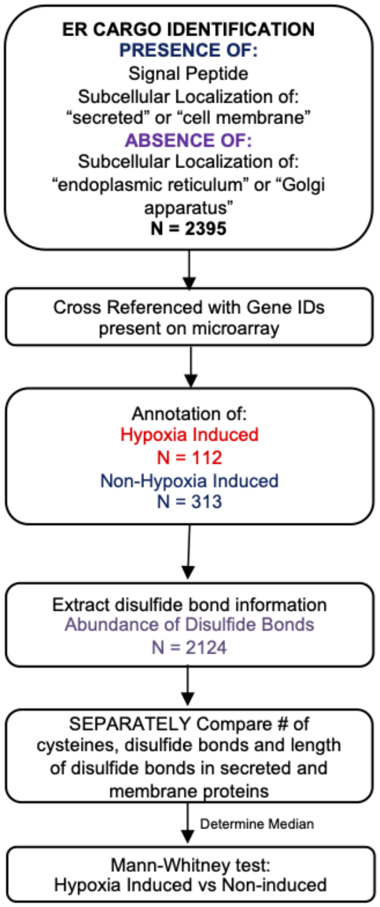

Supporting Information 5. *In silico* analysis pipeline. ER cargo was identified using the Uniprot database by the presence of a signal peptide and an annotated subcellular localization of cell membrane or secreted. These were cross-referenced to microarray data (8) to identify cargo that are >2-fold (n=112) or <1.2 fold (n=313) induced by 24 hours of 0% O_2. Disulfide features for these proteins were extracted from Uniprot. The number of cysteines, disulfide bonds and length of the disulfide bonds were analyzed. Mann-Whitney test was performed between hypoxia-induced and non-hypoxia induced proteins.

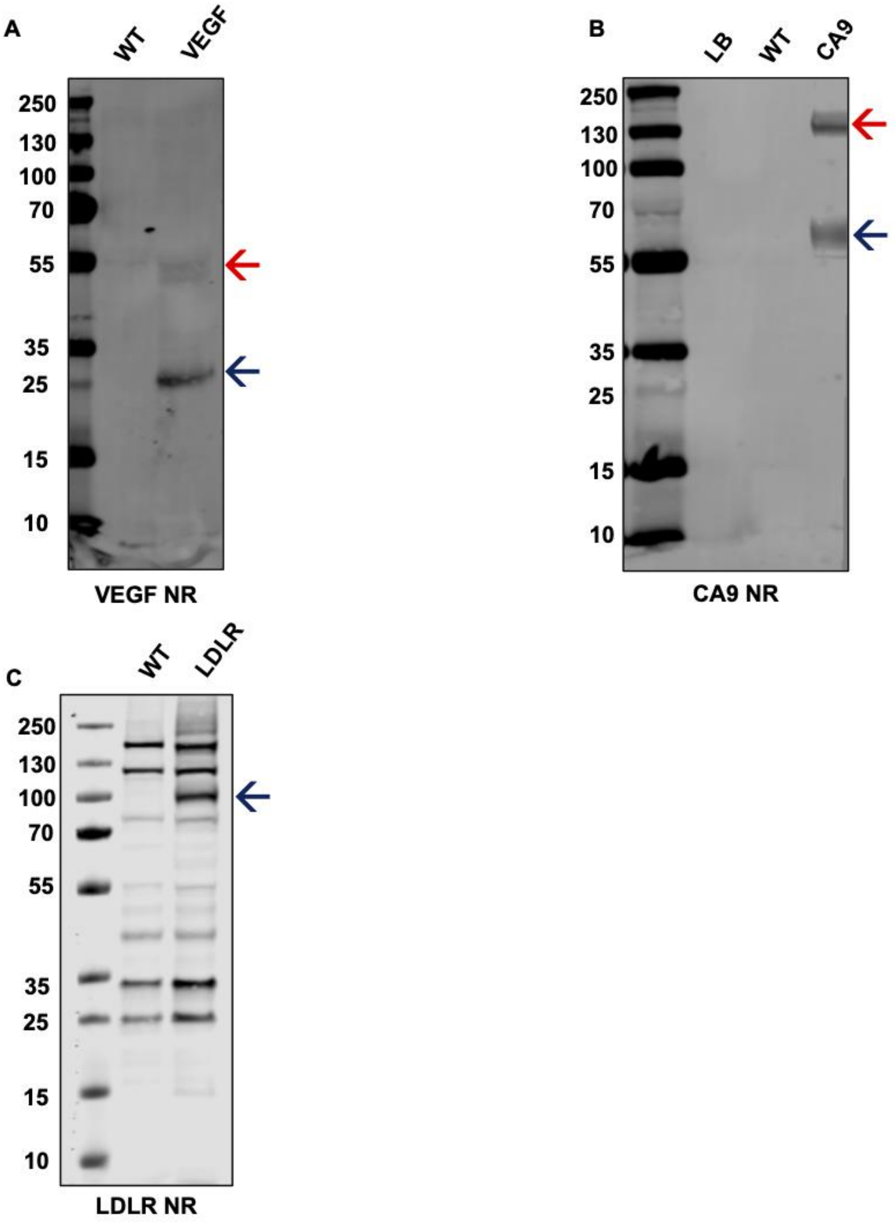

Supporting Information 6. Immunoblots of VEGF-A, CA9 and LDLR. Proteins from HeLa cells transfected with (A) VEGF-A, (B) CA9 and (C) LDLR were resolved under non reducing (NR) conditions prior to western blotting for the same proteins. Arrows represent monomeric (navy) and dimeric (red) bands.
